# What has been the impact of the Traditional Herbal Registration (THR) scheme in the UK on information provided with herbal products bought over the counter?

**DOI:** 10.1186/s12906-019-2494-8

**Published:** 2019-04-11

**Authors:** R. Dickinson, M. C. Kennedy, D. K. Raynor, P. Knapp, M. Thomas, E. Adami

**Affiliations:** 1 School of Health and Community Studies, Leeds Beckett University, Leeds, UK; 2 School of Healthcare, University of Leeds, Leeds, UK; 3 Department of Health Sciences and the Hull York Medical School, University of York, York, UK; 4 School of Languages, Cultures and Societies, Centre for Translation Studies, University of Leeds, Leeds, UK

## Abstract

**Background:**

In 2011 there was a strengthening of European Union (EU) legislation on the licencing of herbal products which, in the UK, resulted in the introduction of the Traditional Herbal Registration (THR) scheme. This scheme sets out standards for the safety and quality of herbal medicines and includes the provision of information to the customer on the safe use of the product.

The aim of this study is to replicate a survey undertaken in 2011, prior to the implementation of the THR scheme, and evaluate the impact of this scheme on the information provided with herbal products bought over-the-counter.

**Methods:**

We undertook a survey on 5 herbal products commonly available over-the-counter (St John’s wort, echinacea, *Ginkgo biloba*, Asian ginseng, garlic). The information was searched for key safety messages identified by the National Center for Complementary and Integrative Health (NCCIH). We also explored the presence of risk of harm information.

**Results:**

We recorded a rise in the number of products registered with the THR scheme (37% in 2016 compared to 7% in 2011). We also identified a reduction in the number of products that did not contain key safety information (75% in 2011 compared to 20% of products obtained in 2016). Risk of harm information was only communicated in products containing a PIL. We identified more products containing frequency of risk of harm information but this was not statistically significant.

**Conclusion:**

The introduction of the THR scheme appears to be associated with an increase in the provision of information about key safety messages on the safe use of herbal products. However, it is important to note that at least half of the products on the market that are not included in the THR scheme do not contain any information about their safe use; this includes information about precautions, interactions and side effects.

The use of NCCIH herbal monographs replicated the methods used in the previous study; we recognise that the use of a different resource might effect the appraisal of the information provided. We also acknowledge that surveying presence of information does not assure that the latter is effectively communicated to patients, for which a close textual analysis would be required. While it is promising that more information is available after the introduction of the THR scheme, the public needs to be informed about ways to optimise safe use of all herbal products.

## Background

There is widespread use of herbal medicines across Europe, America and Australia [1–3]. Herbal products are commonly available in pharmacies, health food shops and supermarkets across the UK. For optimal use of herbal medicines it is important that patients have access to information about their safe and effective use, particularly as the public can perceive herbal medicines as safe despite documented evidence of precautions, interactions and side effects associated with some products [4, 5]. Knowledge of these issues is important for consumers to allow them to make informed decisions about herbal medicines.

However barriers can inhibit the provision of information about the safe and effective use to consumers of herbal medicines. Patients do not always seek information from healthcare professionals about herbal products and do not always disclose their use of them [6]. An ethnographic study of herbal products retailers in the USA suggested there can be variable verbal information provided to consumers at the point of purchase, with the quality of information provided being unreliable and dependent on staff training and expertise [7].

A survey of information provided with herbal products selected from UK pharmacies, supermarkets and health food shops, undertaken by the authors in 2011, found that 75% of products did not contain any information about key issues relevant to safe use [8]. The need for reliable information has been identified as a global priority to enhance the safe and effective use of traditional herbal medicines, and the WHO Traditional Medicine Strategy (2014–2023) sets out a number of strategic global objectives designed to promote and regulate their safe and effective use. Most pertinent are the key objectives which stipulate the need to promote the safety, efficacy and quality of traditional medicine (TM) by expanding the knowledge base, and providing guidance on regulatory and quality assurance standards [9].

The most significant change in regulatory standards in the European Union (EU) over recent years has been the introduction of the European Directive on Traditional Herbal Medicinal Products (2004/24/EC), which sets the registration requirements, that should ensure that herbal medicines meet required standards of quality, safety and evidence of traditional use prior to being available for sale [10]. The EU directive aims to harmonise the definition of traditional herbal medicines across Europe and sets out requirements for the quality and safety of herbal medicines to be assessed prior to licensing. The scheme requires herbal medicines to have a well-documented, consistent, and longstanding use over at least 30 years across Europe and should facilitate consumer access to quality-assured herbal products accompanied with information about their quality and safe use [11].

The directive was enacted in 2004, incorporating a 7 year transition period, meaning that the scheme came into full effect in 2011. By 2011 it was expected that all herbal medicines should have either a THR or a product licence. Prior to the introduction of the THR scheme most available herbal products were ‘unlicensed’ and changes in licencing represent a swing from a market that was largely unregulated, to a regulated one [11]. In our previous study we found that unlicensed products comprised 93% of the sampled products and that there was a significant deficit of good quality information provided on their safe use [12]. It is not clear what the impact of this new scheme has been on the quality of information provided with herbal products, although in 2010 it was noted that the UK had received large numbers of registrations [11].

It has been over 5 years since the introduction of the new regulations and the impact of the THR scheme on the information provided with herbal products is not known. The aim of this study is to replicate the survey undertaken in 2011 and evaluate the impact of the introduction of the THR scheme on the information provided with herbal products bought over the counter. Additionally, we also aim to evaluate and compare the extent to which the information provided with herbal products communicates the likelihood of risk of harm associated with herbal medicines and whether this is in line with recommendations from the UK Medicines and Healthcare products Regulatory Agency (MHRA) and the European Medicines Agency (EMA).

## Methods

### Study design

The study used a survey of a sample collected following same principles as a previous study to undertake a content analysis of the information provided with five herbal products: St John’s wort, garlic, ginkgo, Asian ginseng (Asian) and echinacea. We aimed to replicate the methods of the previous study and these herbal products were collected in the same way as the sample from 2011. The herbal products were chosen as they met one or more of the following criteria:

- There is evidence of drug interaction between the herb and a prescribed medicine [4]
- There exists a published risk-benefit profile on the herb [5]
- The product is available in retail outlets in local shopping areas.

We included all varieties of products containing single herbs i.e. not combined products. The exclusion criteria included products available as creams, liquids, oils, sprays, teas and tinctures. We also excluded combined herbal products.

### Obtaining the products

Products were purchased in 2016 from one UK city. We purchased all the oral use products containing the five herbal products available from the following retailers:

1. Two health food stores: one independent health food store and Holland and Barrett (the UK’s largest retail chain in this sector)
2. Three pharmacies based in supermarkets (Tesco, Sainsbury’s and Asda)
3. Three large chain pharmacies (Boots, Superdrug and Lloyds)

The sample of retailers did not differ significantly from the 2011 survey, however the independent health store used in 2011 has since closed and so a replacement store in the same city was used.

### Evaluation criteria

The study aim was to evaluate the information provided with the herbal products on the completeness and accuracy in communicating key safety issues about the product. We used the US National Center for Complementary and Integrative Health (NCCIH) ‘herbs at a glance’ monographs as the basis for the development of the key safety evaluation criteria, in order to ensure consistency with the methods from the previous survey. The content of each monograph was searched for key safety issues on precautions, interactions and side effects; they were then tabulated resulting in the identification of 16 key points for St John’s wort, 7 for Asian ginseng, 7 for gingko, 6 for garlic and 3 for echinacea. (Table 1).

**Table 1:** Key safety criteria adapted from monographs developed by the National Center for Complementary and Integrative Health (NCCIH)

Herbal product	Precautions	Interactions	Side effects
St John’s Wort	Increased sensitivity to sunlight	Antidepressants Birth control pills Cyclosporin, which prevents the body from rejecting transplanted organs Digoxin, a heart medication Some HIV drugs including indinavir Some cancer medications including irinotecan Warfarin, an anticoagulant Taking ST John’s Wort with certain antidepressants or other drugs that affect serotonin may lead to increased serotonin-related side effects which may be potentially serious	Anxiety Dry mouth Dizziness GI symptoms Fatigue Headache Sexual dysfunction
Ginkgo	If you are older, have a known bleeding risk, or are pregnant you should be cautious about gingko possibly increasing your risk of bleeding	Anticoagulants	Headache Stomach upset Allergic skin reactions
Ginseng (Asian)	Some evidence suggests that ginseng might affect blood sugar and blood pressure. If you have diabetes or high blood pressure consult your healthcare provider before using Asian ginseng Pregnancy and breastfeeding?	warfarin	Headaches Sleep problems Digestive problems
Garlic	Taking garlic may increase the risk of bleeding. If you take an anticoagulant such as warfarin or if you need surgery tell your healthcare provider if you’re taking or planning to take garlic dietary supplements	Warfarin Saquinavir (HIV)	Breath and body odour Heartburn Upset stomach Some people have allergic reactions to garlic
Echinacea	Some people have allergic reactions which might be rare People with atopy may be more likely to have an allergic reaction when taking Echinacea		Digestive tract symptoms such as nausea or stomach pain

In the previous study we identified there was no ‘gold standard’, authoritative source on herbal medicines and so we opted to use the US National Centre for Complementary and Alternative Medicines (NCCAM) herbal monographs as these covered all of the herbal products we had purchased. These herbal monographs are evidence-based resources that provide basic information about specific herbs. Other resources exist, such as the EMA - Community Herbal Monographs but these did not, and do not, contain information about all the sampled herbal products [13]. In 2015 NCCAM become the National Center for Complementary and Integrative Health, or NCCIH. The ‘Herbs at a Glance’ section remains and has been recently updated.

### Data extraction and quality assurance

Data was extracted from the product container (and leaflet, if present) and entered into a Microsoft Excel database according to pre-determined categories. Categories were structured similarly to the Quality Review Document product information template set out by the EMA. Data was extracted by one researcher (RD) and an independent 10% check for accuracy was undertaken by another (MCK).

Agreement on the key safety issues was undertaken as a team (2 pharmacists and 2 nurses agreed the final evaluation criteria as developed from NCCIH). These were tabulated and the original packaging searched for completeness and accuracy. Another 10% check for accuracy was undertaken (MCK).

## Results

### Nature of the products

We found 67 products at 8 different retailers: 21 garlic, 9 St John’s wort, 17 echinacea, 10 *Ginkgo biloba* and 10 Asian ginseng.

### Regulatory category

39.7% (*n* = 25) of the products were THR registered, this demonstrates a large increase in the number of licenced products available on the market, from the situation in 2011 when just 7% of the herbal products (*n* = 5) were licensed (Chi^2^ = 19.4; df = 1; *p* = 0.000011)

One echinacea product did not have evidence of THR registration on its packaging. However it is registered under the scheme. It is possible this is old stock, however for the purposes of the study it have been classed as OTHER *n* = 1 [1.6%].

### Information provided

43% (*n* = 27) of the sample included a leaflet, although the content of two of these largely consisted of promotional materials, rather than information related to the product’s safe and effective use. Registration with the THR registration scheme was associated with an increased likelihood of the presence of a leaflet. Only one product registered under the THR scheme did not include a leaflet. Registered products were more likely to be provided with a leaflet (Chi^2^ = 51.4; df - 1; *p* < .0001).

This represents an increase in the number of products accompanied by a leaflet since the 2011 survey: from 7% in 2011 to 43% in 2016 (Chi^2^ = 22.3; df = 1; *p* = 0.000002).

### Key points of safety information

Tables 2,3,4,5 and 6 show each product, where it was purchased, its licensed usage, and the number of key points of safety information included with the product.

**Table 2:** St John’s wort

Herbal product: SJW	7	8	19	20	45	46	47	58	67
Bought from	Ph	Ph	HF	HF	Ph	Ph	Ph	SM	SM
Legal category	THR	THR	THR	THR	THR	THR	THR	THR	THR
Leaflet supplied	Yes	Yes	Yes	Yes	Yes	Yes	Yes	Yes	Yes
Precautions	2/2	2/2	2/2	2/2	2/2	2/2	2/2	2/2	2/2
Interactions	7/7	7/7	7/7	7/7	7/7	7/7	7/7	7/7	7/7
Side effects	5/7	5/7	5/7	5/7	5/7	5/7	5/7	5/7	5/7
TOTAL XX/16	14/16	14/16	14/16	14/16	14/16	14/16	14/16	14/16	14/16

*Ph* pharmacy, *HF* health food store, *SM* supermarket, *THR* Traditional Herbal Registration, *U* unregulated, *O* other

**Table 3:** Ginkgo

Herbal product: Ginkgo	1	31	32	33	34	35	36	48	51	57
Bought from	Ph	HF	HF	HF	HF	HF	HF	Ph	SM	SM
Legal category	U	U	U	U	U	U	U	U	U	U
Leaflet supplied	No	No	No	No	No	No	No	No	No	No
Precautions	1/3	1/3	0/3	0/3	1/3	1/3	1/3	1/3	1/3	1/3
Interactions	0/1	0/1	0/1	0/1	0/1	0/1	0/1	0/1	0/1	0/1
Side effects	0/3	0/3	0/3	0/3	0/3	0/3	0/3	0/3	0/3	0/3
TOTAL: XX/7	1/7	1/7	0/7	0/7	1/7	1/7	1/7	1/7	1/7	1/7

*Ph* pharmacy, *HF* health food store, *SM* supermarket, *THR* Traditional Herbal Registration, *U* unregulated, *O* other

**Table 4:** Asian ginseng

Herbal product: Asian ginseng	4	21	49	52	60	62
Bought from	Ph	HF	Ph	SM	SM	HF
Legal category	U	U	U	U	U	U
Leaflet supplied	No	No	No	No	No	No
Precautions	3/3	0/3	1/3	1/3	1/3	1/3
Interactions	0/1	0/1	1/1	0/1	0/1	0/1
Side effects	0/3	0/3	0/3	0/3	0/3	0/3
TOTAL: XX/7	3/7	0/7	3/7	1/7	1/7	1/7

*Ph* pharmacy, *HF* health food store, *SM* supermarket, *THR* Traditional Herbal Registration, *U* unregulated, *O* other

**Table 5:** Echinacea

Herbal product: Echinacea	5	6	25	26	27	28	29	30	42	43	44	53	54	56	61	65	66
Bought from	Ph	Ph	HF	HF	HF	HF	HF	HF	Ph	Ph	Ph	SM	SM	SM	SM	HF	Ph
Legal category	THR	THR	THR	THR	O	THR	THR	THR	THR	THR	THR	THR	THR	THR	THR	THR	THR
Leaflet supplied	Yes	Yes	Yes	Yes	Yes	Yes	Yes	Yes	Yes	Yes	Yes	Yes	Yes	Yes	Yes	No	Yes
Precautions	2/2	2/2	2/2	2/2	2/2	2/2	2/2	2/2	2/2	2/2	2/2	2/2	2/2	2/2	2/2	1/2	2/2
Interactions	0/0	0/0	0/0	0/0	0/0	0/0	0/0	0/0	0/0	0/0	0/0	0/0	0/0	0/0	0/0	0/0	0/0
Side effects	0/1	0/1	0/1	0/1	0/1	0/1	0/1	0/1	0/1	0/1	0/1	0/1	0/1	0/1	0/1	0/1	0/1
TOTAL: X/3	2/3	2/3	2/3	2/3	2/3	2/3	2/3	2/3	2/3	2/3	2/3	2/3	2/3	2/3	2/3	1/3	2/3

*Ph* pharmacy, *HF* health food store, *SM* supermarket, *THR* Traditional Herbal Registration, *U* unregulated, *O* other

**Table 6:** Garlic

Herbal product: Garlic	2	3	9	10	11	12	13	14	15	16	17	18	37	38	39	40	41	50	55	59	64
Bought from	Ph	Ph	HF	HF	HF	HF	HF	HF	HF	HF	HF	HF	Ph	Ph	Ph	Ph	Ph	SM	SM	SM	HF
Legal category	U	U	U	U	U	U	U	U	U	U	U	U	U	U	U	U	U	U	U	U	U
Leaflet supplied	No	No	No	No	No	No	No	No	No	No	No	Yes	No	No	No	No	Yes	No	No	No	No
Precautions	0/2	0/2	0/2	0/2	0/2	0/2	0/2	0/2	0/2	0/2	0/2	0/2	0/2	0/2	0/2	0/2	0/2	0/2	0/2	0/2	0/2
Interactions	0/2	0/2	0/2	0/2	0/2	0/2	0/2	0/2	0/2	0/2	0/2	0/2	1/2	1/2	1/2	0/2	0/2	0/2	0/2	0/2	0/2
Side effects	0/4	0/4	0/4	0/4	0/4	0/4	0/4	0/4	0/4	0/4	0/4	0/4	0/4	0/4	0/4	0/4	0/4	0/4	0/4	0/4	0/4
TOTAL: XX/8	0/8	0/8	0/8	0/8	0/8	0/8	0/8	0/8	0/8	0/8	0/8	0/8	1/8	1/8	1/8	1/8	0/8	0/8	0/8	0/8	0/8

*Ph* pharmacy, *HF* health food store, *SM* supermarket, *THR* Traditional Herbal Registration, *U* unregulated, *O* other

We found that both Echinacea and St John’s wort consistently reported some of the key points of safety information. For example, for St John’s wort we identified 16 key points of safety information (2 points of information relating to precautions, 7 to interactions and 5 for side effects). All the St John’s wort products we identified communicated information about 14 out of the 16 key points of safety information.

The remaining products, garlic, gingko and Asian ginseng, included examples of products which did not communicate any key points of safety information. For example, for garlic we identified 8 key points of information (2 points of information relating to precautions, 2 to interactions and 4 to side effects). We found 21 garlic products, 4 of these communicated 1 out of 4 of the key points of safety information and 17 did not contain any of the key points of information.

Table 7 shows the presence or absence of information points for each category of herbal product. In contrast to the 2011 sample, where 75% (*n* = 51) of the sample contained none of the key safety information the 2017 survey demonstrates a large decrease in the products without key safety messages, key safety messages were now included with 68% (*n* = 43) of the products (Chi^2^ = 24.6; df = 1; *p* < 0.000001). All of the products purchased in 2017 with a THR registration contained at least some of the key safety messages compared to around half of the products without a THR (46%, *n* = 17).

**Table 7:** Presence or absence of information points for each category of herbal product

Herbal product	Some points of information	No points of information
Echinacea, *n* = 17 (%)	17 (100%)	0 (0%)
St John’s Wort, *n* = 9 (%)	9 (100%)	0 (0%)
Garlic, *n* = 21 (%)	4 (19%)	17 (81%)
Gingko, *n* = 10 (%)	8 (80%)	2 (20%)
Asian ginseng, *n* = 6 (%)	5 (84%)	1 (16%)
Total, *n* = 63 (%)	43 (68%)	20 (32%)

### Risk of harm information

In PILs for regulated medicines, the risk of harm, or side effect, information is typically presented using both verbal and numerical frequency descriptors [14]. The MHRA recommends that verbal descriptors of risk should be accompanied by corresponding frequency information (e.g. “*Common* (affects *less than 1 in 10 people*)”). We also evaluated provision of such recommended side effect frequency information, comparing the 2011 and 2017 samples, to examine the extent to which herbal products contain information about risk of harm.

For all products, from both 2011 and 2017, risk of harm information was only presented in the context of a patient information leaflet. In 2011 we identified 68 products, 9 of which had product leaflets. Of these nine leaflets, two used a combined format of verbal descriptors and frequency bands. The two leaflets both included the following information:

*“Uncommon side-effects (affecting fewer than 1 in 100 people)”* and *“Other rarer side-effects….”* (St. John’s wort, Karma & Boots)

In 2017 there was an increase in the number of products with accompanying leaflets – 26 out of 63 compared to 9 out of 67 in 2011. We identified that no frequency information was included with products without PILs.

We identified more leaflets reporting frequency information, but this was not statistically significant. In 2017, 15 out of 26 leaflets presented some information of the frequency associated with side effects. This compared to 2 out of 9 leaflets identified in the 2011 sample (Chi^2^ = 3.4; df = 1; *p* = 0.066).

A common approach, in 2017, to reporting frequency information was for PILS to make reference to the frequency associated with risk of harm by stating that the frequency of side effects is not known. See example below:

*“The frequency is not known... This means it is not known how often these reactions occur as there has not been enough reports to allow this information to be calculated.”* Echinacea forte, A Vogel, Boots.

## Discussion

The introduction of the THR scheme for herbal products appears to be associated with an increase in the quality of information provided with herbal products. At least 68% of the products sampled in the updated survey contained some of the points of key information pertinent to the safe use of the product. This compares to 2011 where only a quarter of products contained this information [8].

Our findings also report a significant increase in the number of products registered with the THR scheme, although in 2017 this still represents a minority. As the registration scheme is associated with an increased frequency of information about key safety points then this is a positive move – consumers should be reassured that the THR registered products are usually accompanied with improved and more complete information than those not registered.

For patients to make informed decisions about treatments it is essential that there is full disclosure of any key safety issues associated with taking an herbal medicine. Some herbal products are associated with significant drug interactions and side effects [5, 15]. St John’s wort, for example, has a long documented interaction with cyclosporine, a drug used to prevent organ rejection after transplant [16, 17]. However, our previous study demonstrated that 27% of the St John’s wort products contained no points of key safety information [12]. The recent findings reported here demonstrate that there has been a significant improvement with all the sampled St John’s wort products containing at least some of the key safety information identified.

There are a number of St John’s wort and echinacea products which are registered as THRs, others, such as garlic, may be regarded to be within the definition of a medicine, but may also be available as food supplements. Our findings show that registered medicinal products are also more likely to be provided with key safety information, compared to products lacking compulsory registration. Consumers and healthcare professionals should be made aware of the THR licencing scheme and its association with the increased provision of safety information.

We found one product which was an exception; our sample contained 1 echinacea product which was not THR registered. It is possible that this is old stock, however as the transition period ended in 2011 it is likely to be an unlicensed medicine. It is concerning that products without the required licence are available for purchase over the counter. This was an issue we raised in our 2011 paper and is something that continues to need addressing. We are advised by MHRA that, where complaints are received regarding the sale of borderline medicinal products, they will review products case by case and take appropriate action to remove products that fall within the definition of a medicines from sale [18–20]. There also remains the issue of online availability of products from suppliers outside the jurisdiction of the MHRA [12]; consumers need to receive current information which supports informed and safe use however and wherever they buy a product [21, 22].

Our findings report an increase in the use of MHRA-approved risk descriptors. However, despite an increased willingness to present information about the risks associated with medicines, the incidences of many side effects are not known. This may reflect that such events are rare, generated by isolated reports, or where the absolute risk rate is not known or cannot be quantified, due to a lack of randomised controlled trials of sufficient size.

There is evidence that people tend not to report adverse effects of herbal medicines, or they report them differently to conventional medicines [23, 24]. This also has an impact on pharmacovigilance as accurate reports of side effects and/or drug interactions might not be reported in the first place, and consequently not included in patient information leaflets, which are dependent on post-licencing reporting. Both factors impact on the availability of accurate frequency information about side effects.

It is also widely acknowledged that there is a lack of research data on the safety and effectiveness of herbal medicines which also results in challenges in effectively communicating the risk of harm to patients. The WHO acknowledges that despite a growing interest in Traditional and Chinese medicines (under which herbal medicines are included), there are still many questions about the quality and quantity of evidence to support their use [9]. Indeed, the THR registration scheme does not evaluate the effectiveness of herbal products; it states “No clinical tests and trials on safety and efficacy are required as long as sufficient safety data and plausible efficacy are demonstrated” [25]. Products registered with the THR scheme must have been used for at least 30 years, including at least 15 years in the EU, however there is no requirement for the product to have proven clinical effectiveness prior to registration. Despite the introduction of the THR scheme, which has signified an increase in the quality of information provided to consumers, there remains an information deficit for consumers as a consequence of a lack of clinical data reporting the effectiveness of herbal products.

### Strengths and limitations

We used an updated version of the NCCIH herbal monographs in an attempt to replicate methods from the previous study, although we acknowledge that the use of different resource might have impacted on the evaluation. The criteria generated from the NCCIH monographs were not exhaustive, for example a key safety issue associated with Echinacea is “Digestive tract symptoms such as nausea or stomach pain” [26]. However the leaflets provided with Echinacea did not contain details about this particular side effect, but they did contain very detailed information on the possibility of autoimmune conditions as a side effect [27], something not highlighted by the National Center for Complementary and Integrative Health. This detail was not captured in our analysis.

We sampled a relatively small number of herbal products, and so the information provided with them might not be typical of the sector. Similarly the statistical analysis of the change in provision between 2011 and 2017 is based on small numbers, and so is vulnerable to sampling variation. We acknowledge our sample did not include products such as creams, liquids and tinctures and our findings may not be applicable to these products.

We also acknowledge that the presence of information does not assure that it is effectively communicated to patients, for which a close textual analysis would be required.

## Conclusion

There has been a large increase in the availability of key safety information provided with herbal products in the UK over the period 2011–2017, which may be attributable to the introduction of the THR scheme. However, many herbal products are not included in the THR scheme and at least half of those do not contain any information about safe use (such as about precautions, interactions and side effects). The public needs to be better informed about ways to optimise safe use of all herbal products. As products with registered with the THR scheme have been assessed for quality, safety and traditional use, healthcare professionals could optimise safe use of herbal products by advising on the availability of THR products.

## Abbreviations

EMA: European Medicines Agency
EU: European Union
MHRA: Medicines and Healthcare Products Regulatory Agency
NCCAM: National Centre for Complementary and Alternative Medicine
NCCIH: National Centre for Complementary and Integrative Health
THR: Traditional Herbal Registration
WHO: World Health Organisation
